# Elevated CO_2_ and O_3_ alter the feeding efficiency of *Acyrthosiphon pisum* and *Aphis craccivora* via changes in foliar secondary metabolites

**DOI:** 10.1038/s41598-018-28020-w

**Published:** 2018-07-02

**Authors:** Hongyu Yan, Honggang Guo, Erliang Yuan, Yucheng Sun, Feng Ge

**Affiliations:** 1 State Key Laboratory of Integrated Management of Pest and Rodents, Institute of Zoology, Chinese Academy of Sciences, Beijing, 100101 China; 2 College of Life Sciences, University of Chinese Academy of Sciences, Beijing, 100039 China

## Abstract

Elevated CO_2_ and O_3_ can affect aphid performance via altering plant nutrients, however, little is known about the role of plant secondary metabolites in this process, especially for aphids feeding behaviors. We determined the effects of elevated CO_2_ and O_3_ on the growth and phenolics of alfalfa (*Medicago sativa*) and feeding behaviors of the pea aphids (*Acyrthosiphon pisum*) and cowpea aphids (*Aphis craccivora*). Elevated CO_2_ improved plant growth, but could not completely offset the negative effects of elevated O_3_. Elevated O_3_ increased foliar genistin content at the vegetative stage, increased ferulic acid at the reproductive stage, and elevated CO_2_ increased those at both stages. Simultaneously elevated CO_2_ and O_3_ increased foliar ferulic acid content at the reproductive stage and increased genistin content at both stages. For pea aphids, feeding efficiency was reduced under elevated CO_2_ at the reproductive stage and decreased under elevated O_3_ at the vegetative stage. For cowpea aphids, feeding efficiency was increased under elevated CO_2_ at the vegetative stage and decreased under elevated O_3_ at both stages. Simultaneously elevated CO_2_ and O_3_ decreased both aphids feeding efficiency. We concluded that CO_2_ and O_3_ independently or interactively had different effects on two aphids feeding behaviors through altering foliar ferulic acid and genistin contents.

## Introduction

Global atmospheric concentrations of greenhouse gases (e.g., CO_2_ and O_3_) have increased due to human activities since industrialization. The CO_2_ concentration has increased from 280 μL/L to approximately 400 μL/L in 2017 (https://www.co2.earth/), and the tropospheric O_3_ concentration has increased from 10 nL/L to 50 nL/L in 2009^1^. Furthermore, the concentrations of CO_2_ and O_3_ are expected to continue to increase^2^. Increases in CO_2_ and O_3_ concentrations have been anticipated to greatly influence agricultural and forest ecosystems^3,4^; they can directly affect plant growth, primary and secondary metabolisms, and indirectly alter interactions between plants and herbivorous insects^5–7^.

Elevated CO_2_ typically stimulates plant growth, decreases plant nitrogen concentrations, and increases the carbon:nitrogen (C:N) ratio^4,8^. Conversely, O_3_, as an oxidizing agent, enters the leaf interior through the stomata and causes leaf tissue damage, thereby inhibiting plant growth^9^; but the responses of foliar nutrients to elevated O_3_ are species-specific and depend on the duration of O_3_ exposure^10^. Elevated CO_2_ and O_3_ generally increase plant secondary metabolites, such as phenolics, including total phenolics, condensed tannins, and flavonoids^6,7,11^, and they also interactively affect plant metabolism that elevated CO_2_ tends to offset the induction of phenolics by elevated O_3_^6,12^. In addition, the plant chemical composition and concentrations often change with ontogenetic stage^13^. For example, phenolic acids, i.e., trans-2-hydroxycinnamic, rosmarinic, vanillic, chlorogenic, gallic, and cinnamic acids, dominate during the early vegetative stage; whereas flavonoids, including amentoflavone, apigenin, quercetin, luteolin, coumarin, and rutin, predominate during the other growth stages in sweet marjoram (*Origanum majorana*)^14^. Furthermore, the concentrations of glucosinolates in *Arabidopsis thaliana* and phenolic glycoside in trembling aspen (*Populus tremuloides*) are higher in the younger leaves than in the older leaves^15,16^. The plant ontogenetic stage and climate changes may interactively influence plant secondary metabolites, for example, phenolic glycoside concentrations increase in the leaves of the younger trees but decrease in the older trees under elevated CO_2_, while elevated O_3_ has the opposite effects^17^. These changes in plant primary and secondary metabolites under elevated CO_2_ and O_3_ in turn influence the performance of insect herbivores^6,7^.

Many previous work has shown that elevated CO_2_ or O_3_ reduce the performance of most leaf-chewing insects due to decreased nitrogen concentrations in plants^7,18,19^. On the other hand, the increased concentration of plant secondary metabolites may partially explain the rduced performance of leaf-chewing insects under elevated CO_2_ and O_3_, because the secondary compounds can induce or decline the detoxication activities of the digestion system, which thereby prolonging developmental time and reducing growth rates^20,21^. For the piercing-sucking insects e.g., aphids, many studies have shown that elevated CO_2_ or O_3_ can increase aphid performance via promoting plant nitrogen based nutrition^22–25^. However, Johnson *et al*.^26^ find that the fecundity of the pea aphid (*Acyrthosiphon pisum*) responds differently to elevated CO_2_ when fed on five different alfalfa (*Medicago sativa*) cultures. Furthermore, the population abundance of *Rhopalosiphum padi* is reduced under elevated CO_2_ when fed on tall fescue (*Schedonorus arundinaceus*), but is increased when fed on barley (*Hordeum vulgare*)^27^. These results presumably indicate that the same aphid species that fed on different host plants exhibits different responses to climate changes. On the other hand, different aphid species or even genotypes that fed on the same host plants may also perform differentially under elevated CO_2_ and O_3_^28^. For example, the specialist *Brevicoryne brassicae* are larger and accumulate more fat, while no changes are found in the generalist *Myzus persicae* reared on *Brassica oleracea* under elevated CO_2_^29^. Therefore, the responses of aphids to elevated CO_2_ and O_3_ seem to be species-specific, demonstrating decreased, increased, or unchanged population abundance, growth, and fecundity^30–33^, but more evidence is needed to explain the heterogeneous responses.

Plant secondary metabolites may also contribute to the idiosyncratic responses of aphids to climate changes, though few studies have focused on it^34,35^. For the aphids that feed exclusively on the phloem sap, plant secondary metabolites mainly affect aphid feeding behaivor rather than the digestion system because the secondary metabolites rarely distribute in the phloem sap^36,37^. Many plant secondary compounds, including alkaloid, luteolin, genistein, apigenin, and saponin etc., can negatively affect the penetration pathway stage of aphid feeding^38–41^. For example, caffeic, ferulic, and sinapic acids disfavor the grain aphid (*Sitobion avenae*) feeding by prolonging the early pathway phases of probing, increasing the number of probing, and reducing salivation into sieve elements and ingestion of phloem sap^42^. Furthermore, the same secondary metabolites seem to have different effects on feeding activities of different aphid species. For example, the total time of probing of *Aphis fabae*, *Aphis craccivora*, *A*. *pisum*, and *M*. *persicae* increases with a reduced alkaloid content in narrow-leafed lupins (*Lupinus angustifolius*), whereas the alkaloid content has no influence on *Macrosiphum albifrons*; furthermore, when fed on the alkaloid-rich cultivar ‘Azuro’, a reduced occurrence of phloem phases is observed, especially for *A*. *pisum* and *A*. *fabae*, whereas *M*. *albifrons* shows the longest phloem phase^38^. However, it is also reported that secondary compounds may act as probing stimulants of aphids^43^. Therefore, the increases in plant secondary metabolites may increase or decrease epidermis and mesophyll resistance against aphids during pathway and probing feeding stages under elevated CO_2_ or O_3_, and the differential feeding responses of aphid species to plant secondary metabolites might contribute to their idiosyncratic responses to elevated CO_2_ or O_3_. However, the evidence about how climate changes, especially for elevated O_3_ alter the aphid feeding activities via plant secondary metabolites is lacking.

The current study aimed to investigate how elevated CO_2_ and O_3_, alone or in combination, alter plant secondary metabolites at different ontogenetic stages and produce cascading effects on aphid feeding behaviors. By using 12 field open-top chambers with four treatments (control, elevated CO_2_, elevated O_3_, and elevated CO_2_ and O_3_), we measured plant growth traits, secondary metabolites, and feeding behaviors of the pea aphid (*Acyrthosiphon pisum* Harris) and the cowpea aphid (*Aphis craccivora* Koch) on alfalfa (*Medicago sativa*). Our specific objectives were to determine: (1) the effects of elevated CO_2_ and O_3_ on plant growth traits and secondary metabolites phenolics of alfalfa, and (2) the feeding behaviors of the two species of aphids when fed on alfalfa grown under different treatments.

## Results

### Responses of plant growth traits to elevated CO_2_ and O_3_

**Figure 1:**
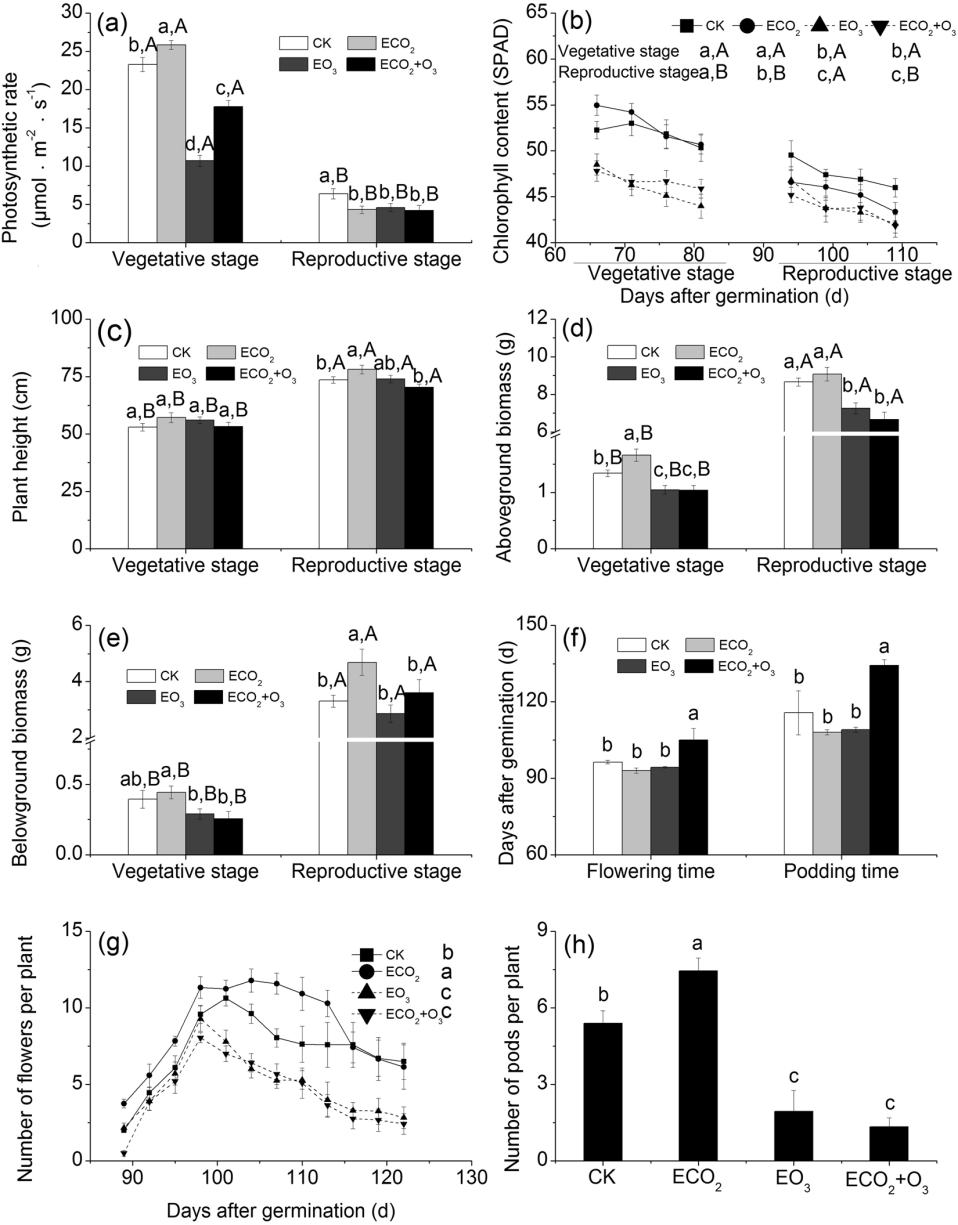
Growth traits of alfalfa grown under ambient or elevated CO_2_ and O_3_. (**a**) Photosynthetic rate, (**b**) Chlorophyll content, (**c**) Plant height, (**d**) Aboveground biomass, (**e**) Belowground biomass, (**f**) Flowering time and podding time, (**g**) Number of flowers per plant, and (**h**) Number of pods per plant. Values are the mean (±SE) of three replicates. Different lowercase letters indicate significant differences among the CO_2_ and O_3_ treatments within the same plant developmental stage. Different uppercase letters indicate significant differences between developmental stages within the same CO_2_ and O_3_ concentrations.

Elevated CO_2_, O_3_, plant developmental stage, and their interactions significantly influenced alfalfa growth (Table 1). Elevated CO_2_ did not significantly alter the plant chlorophyll content but did increase the plant net photosynthetic rate at the vegetative stage, while decreased them at the reproductive stage (significant CO_2_ × developmental stage interaction, Table 1, Fig. 1a,b). Elevated CO_2_ increased plant aboveground biomass only at the vegetative stage (Fig. 1d) and increased plant belowground biomass only at the reproductive stage (Fig. 1e). Elevated CO_2_ also increased the numbers of flowers and pods (Fig. 1g,h). Contrary to elevated CO_2_, O_3_ fumigation decreased the plant chlorophyll content, net photosynthetic rate, and aboveground biomass at both developmental stages, and decreased plant belowground biomass at the vegetative stage, regardless of CO_2_ levels (Table 1, Fig. 1a,b,d and e). Furthermore, O_3_ fumigation alone or in combination with CO_2_, also decreased the numbers of flowers and pods (Fig. 1g,h). Neither elevated CO_2_ nor elevated O_3_ affected plant flowering and podding time, though simultaneously elevated CO_2_ and O_3_ significantly delayed both (Table 1, Fig. 1f). The plant height was only significantly influenced by developmental stage, with significantly higher plant height at the reproductive stage than that at the vegetative stage (Table 1, Fig. 1c).

**Table 1:** Effects of CO_2_, O_3_, plant developmental stage, and their interactions on alfalfa growth traits. *F* and *P* values from MANOVAs are shown.

Plant growth traits	CO_2_		O_3_		Developmental stage		CO_2_ × O_3_		CO_2_ × developmental stage		O_3_ × developmental stage		CO_2_ × O_3_ × developmental stage	
	*F*	*P*	*F*	*P*	*F*	*P*	*F*	*P*	*F*	*P*	*F*	*P*	*F*	*P*
Photosynthetic rate	12.416	**0**.**001**	122.083	**<0**.**001**	807.924	**<0**.**001**	17.667	**<0**.**001**	34.458	**<0**.**001**	85.638	**<0**.**001**	3.216	0.076
Chlorophyll content	0.070	0.792	19.599	**<0**.**001**	25.991	**<0**.**001**	1.801	0.182	2.446	0.120	2.658	0.105	0.121	0.728
Plant height	0.913	0.341	1.006	0.317	218.391	**<0**.**001**	11.098	**0**.**001**	0.002	0.966	2.680	0.104	0.003	0.958
Aboveground biomass	0.001	0.975	50.332	**<0**.**001**	1477.290	**<0**.**001**	3.541	0.062	0.418	0.519	18.135	**<0**.**001**	1.124	0.291
Belowground biomass	8.674	**0**.**004**	7.223	**0**.**008**	296.733	**<0**.**001**	0.664	0.417	7.778	**0**.**006**	3.179	0.077	0.358	0.551
Flowering time	2.301	0.180	4.279	0.084			8.387	**0**.**027**						
Podding time	0.521	0.497	0.646	0.452			1.819	0.226						
Number of flowers per plant	4.126	**0**.**045**	50.080	**<0**.**001**			1.893	0.172						
Number of pods per plant	4.109	**0**.**047**	168.686	**<0**.**001**			13.533	**<0**.**001**						

*P* values < 0.05 are bolded.

### Responses of plant secondary metabolites to elevated CO_2_ and O_3_

**Figure 2:**
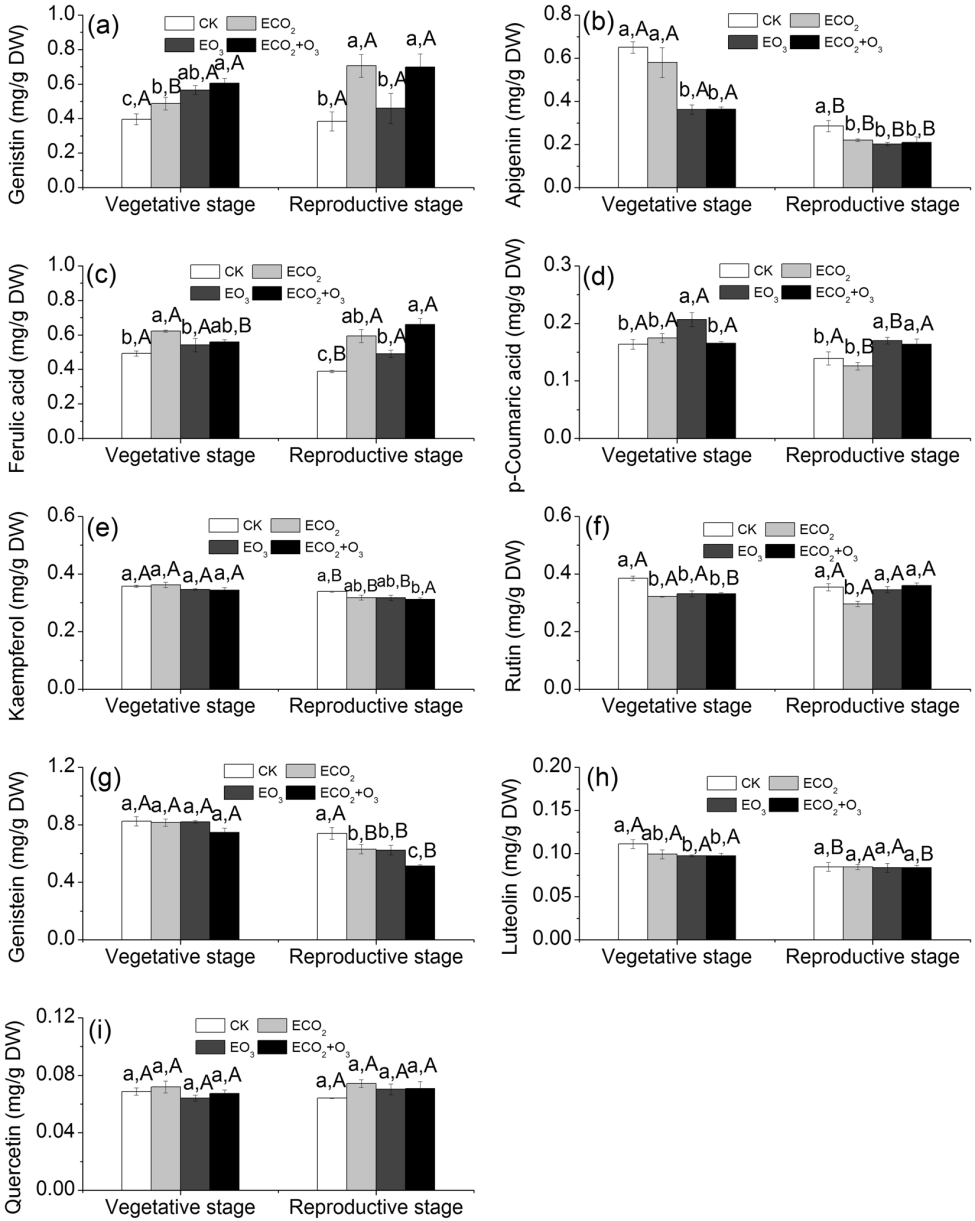
Foliar secondary metabolites contents in alfalfa grown under ambient or elevated CO_2_ and O_3_. (**a**) Genistin, (**b**) Apigenin, (**c**) Ferulic acid, (**d**) *p*-coumaric acid, (**e**) Kaempferol, (**f**) Rutin, (**g**) Genistein, (**h**) Luteolin, and (**i**) Quercetin. Each value represents the average (±SE) of three replicates. Different lowercase letters indicate significant differences among the CO_2_ and O_3_ treatments within the same plant developmental stage. Different uppercase letters indicate significant differences between developmental stages within the same CO_2_ and O_3_ concentrations.

Elevated CO_2_ significantly influenced foliar rutin, genistein, ferulic acid, and genistin concentrations (Table 2). Elevated O_3_ significantly affected foliar kaempferol, apigenin, genistein, *p*-coumaric acid, ferulic acid, and genistin contents (Table 2). Elevated CO_2_ alone increased foliar ferulic acid content at both developmental stages, and elevated O_3_ alone or in combination with CO_2_ increased it only at the reproductive stage (significant CO_2_ × developmental stage interaction, significant O_3_ × developmental stage interaction, Table 2, Fig. 2c). Elevated O_3_ alone increased foliar *p*-coumaric acid content at the vegetative stage, and increased it at the reproductive stage, regardless of CO_2_ levels (Table 2, Fig. 2d). CO_2_ and O_3_ fumigation increased foliar genistin content at the vegetative stage, but only CO_2_ fumigation increased genistin content at the reproductive stage (Fig. 2a). CO_2_ and O_3_ fumigation decreased foliar apigenin content at both developmental stages, with higher values at the vegetative stage than those at the reproductive stage (Table 2, Fig. 2b). Both gases fumigation decreased foliar rutin content only at the vegetative stage and decreased genistein content only at the reproductive stage (significant CO_2_ × developmental stage interaction, significant O_3_ × developmental stage interaction, Table 2, Fig. 2f,g). Overall, elevated CO_2_ and O_3_ had significant effects on alfalfa foliar secondary metabolites, and these effects varied between vegetative stage and reproductive stage.

**Table 2:** Effects of CO_2_, O_3_, plant developmental stage, and their interactions on alfalfa foliar secondary metabolite contents. *F* and *P* values from MANOVAs are shown.

Secondary metabolites	CO_2_		O_3_		Developmental stage		CO_2_ × O_3_		CO_2_ × developmental stage		O_3_ × developmental stage		CO_2_ × O_3_ × developmental stage	
	*F*	*P*	*F*	*P*	*F*	*P*	*F*	*P*	*F*	*P*	*F*	*P*	*F*	*P*
Kaempferol	1.666	0.218	8.618	**0**.**011**	40.404	**<0**.**001**	0.162	0.693	2.099	0.169	0.003	0.954	1.393	0.257
Rutin	7.512	**0**.**016**	4.154	0.061	1.049	0.323	49.999	**<0**.**001**	6.010	**0**.**028**	31.700	**<0**.**001**	3.883	0.069
Apigenin	2.688	0.123	56.624	**<0**.**001**	170.928	**<0**.**001**	3.268	0.092	0.016	0.901	26.736	**<0**.**001**	0.002	0.967
Genistein	19.466	**0**.**001**	19.698	**0**.**001**	103.622	**<0**.**001**	0.975	0.340	3.902	0.068	5.470	**0**.**035**	0.840	0.375
Luteolin	1.302	0.273	2.615	0.128	44.409	**<0**.**001**	1.362	0.263	1.288	0.276	1.662	0.218	1.230	0.286
Quercetin	3.285	0.091	0.475	0.502	0.577	0.460	0.986	0.338	0.221	0.646	1.627	0.223	1.043	0.325
*p*-Coumaric acid	4.403	0.054	16.082	**0**.**001**	19.018	**0**.**001**	4.544	0.051	0.516	0.485	2.102	0.169	6.218	**0**.**026**
Ferulic acid	50.654	**<0**.**001**	4.673	**0**.**048**	1.053	0.322	3.631	0.077	9.938	**0**.**007**	6.503	**0**.**023**	1.288	0.275
Genistin	18.965	**0**.**001**	5.042	**0**.**041**	1.519	0.238	0.713	0.413	7.363	**0**.**017**	1.916	0.188	0.035	0.854

*P* values **< **0.05 are bolded.

### Responses of aphid feeding behaviors to elevated CO_2_ and O_3_

**Figure 3:**
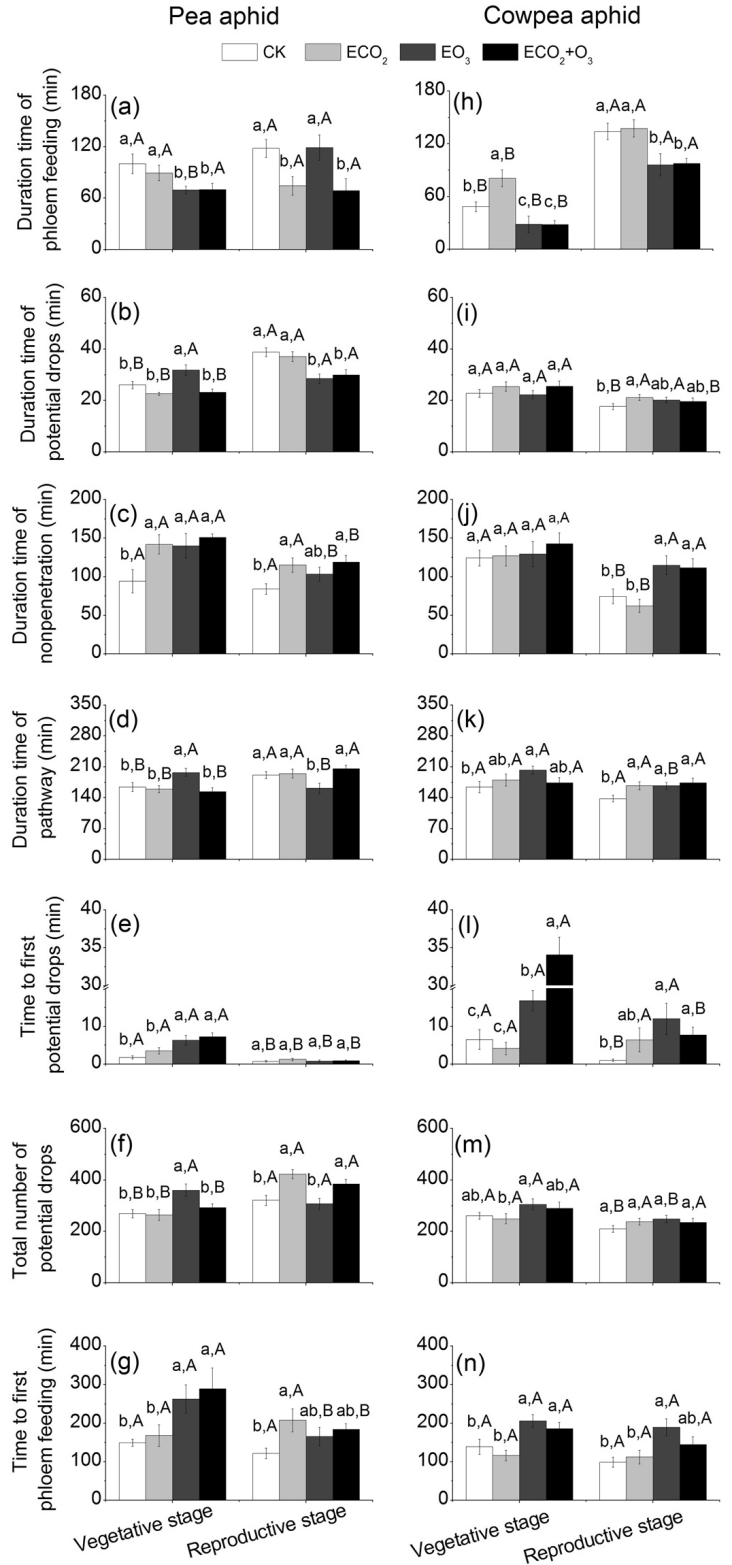
The time pea aphids and cowpea aphids spent in various feeding activities on alfalfa grown under ambient or elevated CO_2_ and O_3_. (**a**–**g**) Various feeding activities of pea aphids, (**h**–**n**) Various feeding activities of cowpea aphids. ‘Phloem feeding’ indicates that aphids are injecting watery saliva into the sieve element and ingesting the phloem sap; ‘potential drops’ indicates that aphids briefly puncture cells during plant penetration; ‘nonpenetration’ indicates that stylets are outside the plants; ‘pathway’ indicates that mostly intramural probing activities between mesophyll or parenchyma cells. Values are the means (±SE) of 21 biological replicates. Different lowercase letters indicate significant differences among the CO_2_ and O_3_ treatments within the same plant developmental stage. Different uppercase letters indicate significant differences between developmental stages within the same CO_2_ and O_3_ concentrations.

Elevated CO_2_ had significant effects on the time the pea aphids spent on nonpenetration, phloem feeding, potential drops, time to first potential drops, and time to first phloem feeding. Elevated O_3_ and developmental stage had effects similar to those for elevated CO_2_, except for phloem feeding (Table 3). Elevated CO_2_ had no significant impacts on the time the pea aphids spent on phloem feeding at the vegetative stage but decreased it at the reproductive stage, whereas elevated O_3_ had the opposite effects (Fig. 3a). Simultaneously elevated CO_2_ and O_3_ reduced the feeding efficiency of the pea aphids at both developmental stages (Fig. 3a), especially at the vegetative stage, simultaneously elevated CO_2_ and O_3_ increased the time to first potential drops and time to first phloem feeding (Fig. 3e,g).

**Table 3:** Effects of CO_2_, O_3_, plant developmental stage, and their interactions on aphid feeding activities. *F* and *P* values from MANOVAs are shown.

Aphids feeding activities	CO_2_		O_3_		Developmental stage		CO_2_ × O_3_		CO_2_ × developmental stage		O_3_ × developmental stage		CO_2_ × O_3_ × developmental stage	
	*F*	*P*	*F*	*P*	*F*	*P*	*F*	*P*	*F*	*P*	*F*	*P*	*F*	*P*
**Pea aphid**														
np	10.507	**0**.**002**	6.724	**0**.**011**	13.375	**<0**.**001**	2.930	0.090	0.126	0.723	1.256	0.265	0.292	0.590
C	0.025	0.876	0.213	0.645	7.913	**0**.**006**	0.101	0.752	11.749	**0**.**001**	2.774	0.098	7.721	**0**.**006**
E	12.509	**0**.**001**	2.149	0.147	3.335	0.072	0.002	0.966	8.202	**0**.**005**	2.412	0.124	0.590	0.445
pd	5.386	**0**.**022**	20.220	**<0**.**001**	29.310	** < 0**.**001**	0.075	0.785	4.199	**0**.**043**	22.566	**<0**.**001**	2.327	0.130
Time to first pd	9.641	**0**.**002**	13.297	**<0**.**001**	112.430	**<0**.**001**	1.474	0.227	4.104	**0**.**045**	38.395	**<0**.**001**	0.064	0.801
Time to first E	4.162	**0**.**044**	8.828	**0**.**004**	3.675	0.058	2.397	0.125	1.936	0.167	8.914	**0**.**004**	0.866	0.354
Total number of pd	7.001	**0**.**009**	0.029	0.864	15.048	**<0**.**001**	2.484	0.117	22.365	**<0**.**001**	7.362	**0**.**008**	0.367	0.546
**Cowpea aphid**														
np	0.057	0.812	12.406	**0**.**001**	24.891	**<0**.**001**	0.385	0.536	0.666	0.416	4.234	**0**.**042**	0.013	0.911
C	1.696	0.195	5.275	**0**.**023**	5.207	0.024	5.395	**0**.**022**	2.644	0.106	0.027	0.870	0.610	0.436
E	3.498	0.065	64.482	**0**.**000**	157.132	**<0**.**001**	3.424	0.067	1.846	0.178	0.152	0.698	1.850	0.177
pd	4.510	**0**.**035**	0.002	0.963	16.261	**<0**.**001**	1.373	0.243	0.485	0.487	0.185	0.668	1.395	0.239
Time to first pd	4.199	**0**.**043**	53.929	**<0**.**001**	18.124	**<0**.**001**	0.014	0.907	2.026	0.158	13.407	**<0**.**001**	18.255	**<0**.**001**
Time to first E	2.531	0.114	19.853	**<0**.**001**	3.129	0.080	1.036	0.311	0.032	0.859	0.088	0.767	1.271	0.262
Total number of pd	0.218	0.641	5.630	**0**.**019**	12.544	**0**.**001**	2.198	0.141	0.948	0.332	0.935	0.335	0.586	0.445

*P* values **< **0.05 are bolded. np (nonpenetration), stylets are outside the plants; C (pathway), mostly intramural probing activities between mesophyll or parenchyma cells; E (phloem feeding), aphids are injecting watery saliva into the sieve element and ingesting the phloem sap; pd (potential drops), aphids briefly puncture cells during plant penetration.

Elevated CO_2_ had little effect on the cowpea aphids feeding, but elevated O_3_ and developmental stage had significantly altered their feeding activities (Table 3). Elevated CO_2_ increased the time the cowpea aphids spent on phloem feeding at the vegetative stage, but had no effects at the reproductive stage (Fig. 3h). Elevated O_3_, alone or in combination with CO_2_, decreased the time the cowpea aphids spent on phloem feeding at both developmental stages (Fig. 3h). O_3_ fumigation also increased the time to first potential drops and time to first phloem feeding of the cowpea aphids at both developmental stages, regardless of CO_2_ levels (Fig. 3l,n).

### Relationships between plant secondary metabolites and aphid feeding activities

Based on the aforementioned results, we calculated correlations between plant secondary metabolites that were significantly altered by CO_2_ and O_3_, and aphids feeding time of phloem feeding and potential drops. The results from the correlation analysis showed that the foliar apigenin and *p*-coumaric acid contents were significantly negatively correlated with the duration time of potential drops of the pea aphids, whereas the foliar ferulic acid and genistin contents were negatively correlated with the duration time of phloem feeding of the pea aphids (Table 4). The foliar *p*-coumaric acid content was significantly negatively correlated with the duration time of phloem feeding of the cowpea aphids, and ferulic acid content was positively correlated with the the duration time of potential drops of the cowpea aphids (Table 4).

**Table 4:** Relationships between secondary metabolite contents in the leaves of alfalfa and the time aphids spent on E (phloem feeding) and pd (potential drops). Correlation coefficient *r* and *P* values are shown.

Secondary metabolites	Pea aphid						Cowpea aphid					
	E			pd			E			pd		
	N	*r*	*P*	N	*r*	*P*	N	*r*	*P*	N	*r*	*P*
Rutin	8	0.167	0.693	8	−0.356	0.387	8	−0.121	0.776	8	−0.352	0.392
Apigenin	8	−0.054	0.900	6	−0.819	**0**.**046**	8	−0.510	0.197	8	0.623	0.099
Genistein	8	0.108	0.800	8	−0.050	0.905	8	−0.552	0.156	8	0.531	0.176
*p*-Coumaric acid	8	−0.232	0.580	7	−0.867	**0**.**012**	8	−0.781	**0**.**022**	8	0.388	0.342
Ferulic acid	8	−0.772	**0**.**025**	8	−0.584	0.128	8	−0.134	0.752	8	0.785	**0**.**037**
Genistin	8	−0.837	**0**.**010**	8	−0.118	0.780	8	0.066	0.877	8	0.105	0.805

Values in bold indicate a significant correlation. E (phloem feeding) indicates that aphids are injecting watery saliva into the sieve element and ingesting the phloem sap; pd (potential drops) indicates that aphids briefly puncture cells during plant penetration.

### Effects of secondary compounds on aphid feeding behaviors

**Figure 4:**
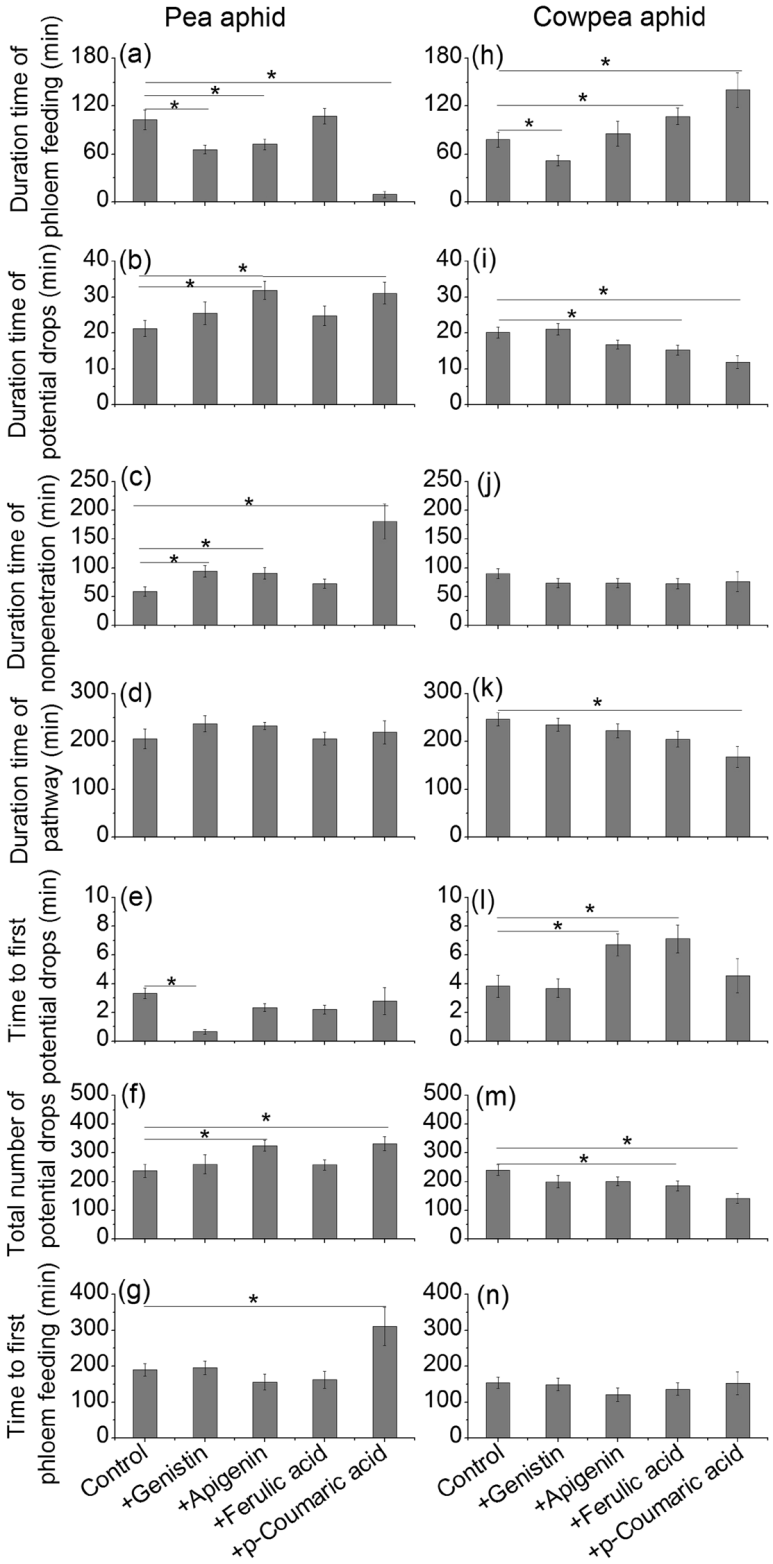
The time pea aphids and cowpea aphids spent in various feeding activities on treated (+genistin, +apigenin, +ferulic acid, and +*p*-coumaric acid) and control plants. (**a**–**g**) Various feeding activities of pea aphids, (**h**–**n**) Various feeding activities of cowpea aphids. ‘Phloem feeding’ indicates that aphids are injecting watery saliva into the sieve element and ingesting the phloem sap; ‘potential drops’ indicates that aphids briefly puncture cells during plant penetration; ‘nonpenetration’ indicates that stylets are outside the plants; ‘pathway’ indicates that mostly intramural probing activities between mesophyll or parenchyma cells. Values are the means (±SE) of 15–20 biological replicates. Significant differences: *there was significant difference between the treatment and control at *P* < 0.05.

The time the pea aphids spent on phloem feeding was significantly reduced on treated plants but not on +ferulic acid compared to controls (Fig. 4a). The time that the pea aphids spent on potential drops was significantly increased, and the total number of potential drops was significantly increased on the plants treated with +apigenin and +*p*-coumaric acid (Fig. 4b,f).

Supplemental genistin also decreased the time the cowpea aphids spent on phloem feeding, whereas +ferulic acid and +*p*-coumaric acid significantly increased it (Fig. 4h). In addition, +ferulic acid and +*p*-coumaric acid reduced the time the cowpea aphids spent on potential drops and the total number of potential drops (Fig. 4i,m).

## Discussion

Elevated CO_2_ and O_3_ affect the performance of herbivorous insects mainly by altering host plant primary and secondary metabolites^6^. Phenolics are important secondary metabolites for plants in defence against herbivory^44^. In the current study, elevated CO_2_ and O_3_ altered alfalfa growth traits and concentrations of phenolics such as genistin, ferulic acid, *p*-coumaric acid, and apigenin, which were antifeedants or feeding stimuli to aphids according to our exogenous application bioassay. Thus, aphid feeding efficiency was differentially altered by these changes in secondary metabolites. Furthermore, the responses of plant secondary metabolites and corresponding aphid feeding activities to elevated CO_2_ and O_3_ varied between plant developmental stages.

The positive effects of elevated CO_2_ on the plant photosynthesis, growth, and seed yield in legume alfalfa are consistent with the results of a previous work that has studied soybean (*Glycine max*)^45^. However, elevated O_3_ negatively affected alfalfa growth, and these reductions in plant photosynthesis and growth under elevated O_3_ may be due to the generation of ROS damage to photosynthetic processes such as the synthesis of rubisco^46^. Our study also showed that elevated CO_2_ did not offset the negative effects of elevated O_3_ on the plant growth. This finding is in contrast to other studies in which elevated CO_2_ has been shown to ameliorate the negative effects of elevated O_3_^47,48^. These contradictory results may be due to the interactions between CO_2_ and O_3_ depending on plant species^49^ and plant developmental stages^17^.

In addition to having effects on alfalfa growth traits, elevated CO_2_ and O_3_ altered the concentrations of the foliar secondary metabolites phenolics, such as rutin, ferulic acid, genistin, apigenin, and *p*-coumaric acid. For example, ferulic acid and genistin contents were significantly increased at both plant developmental stages under elevated CO_2_. Plant phenolics are formed from phenylalanine via the shikimic acid-phenylpropanoid pathway^50^, and the biosynthesis of phenylpropanoids requires the efficient flow of carbon into phenylalanine biosynthesis^51^. This process can be regulated by phytohormones, such as JA (jasmonic acid), SA (salicylic acid), and ET (ethylene), which can be affected by elevated CO_2_^52,53^. For example, elevated CO_2_ increases the concentration of SA-regulated phenolics, such as flavonoids (e.g., quercetin, kaempferol, and fisetin) in Malaysian young ginger (*Zingiber officinale* Roscoe)^54^, but reduces the concentration of JA-regulated isoflavonoids, such as genistein in soybean (*G*. *max*)^55^. Although our results are not absolutely consistent with these studies mentioned above, we all indicate that individual phenolic compounds differentially respond to elevated CO_2_. In addition, plant secondary metabolites, such as alkaloids and phenolics, often change dramatically as plants develop^56,57^. Furthermore, plant developmental stages interact with atmospheric changes to influence the secondary metabolism^6^. However, most studies have investigated only one developmental stage. For example, six of the nine sympatric British grassland species studied exhibits a significant increase in one or more secondary metabolites throughout seedling ontogeny^56^. Few studies have focused on multiple ontogenetic stages, such as the seedling stage, vegetative juvenile stage, and mature stage^58^. Our study included two plant developmental stages, the vegetative and reproductive stages. We found that the responses of foliar ferulic acid and genistin contents to elevated CO_2_ and O_3_ were influenced by the plant developmental stage. For example, genistin content was increased only at the vegetative stage, ferulic acid content was increased only at the reproductive stage under elevated O_3_, and the foliar apigenin, kaempferol, and genistein contents were much higher at the vegetative stage than those at the reproductive stage. The heterogeneous defence chemical composition between vegetative and reproductive stages might be explained by that the direction of changes in defensive compounds during the transition from juvenile to mature stage depends on the types of compounds in herbs^58^. An increase in phenolic content under O_3_ fumigation is also commonly reported^6^, though elevated O_3_ has been shown to damage plant photosynthesis^59^. These changes in phenolics under elevated O_3_ may be due to the increase in the activities of phenylalanine-ammonium lyase (PAL) and chalcone synthase enzymes (CHS), which are key enzymes in the biosynthesis of phenolics^60^. The increased phenolics may also act as antioxidants against oxidative stress caused by O_3_^61^.

Plant secondary metabolites can affect the behavior, growth, and development of herbivorous insects^62,63^. According to our bioassay, ferulic acid and *p*-coumaric acid acted as feeding stimulants of the cowpea aphids, while ferulic acid did not affect the pea aphids, and *p*-coumaric acid acted as an antifeedant of the pea aphids. These results suggest that a single compound can have different and even opposite effects on these two species of aphids. Similar results have been reported for *p*-coumaric acid and ferulic acid, which are phagostimulants for the stem borer (*Chilo partellus* Swinhoe)^64^, but feeding inhibitors for maize weevil (*Sitophilus zeamais* Motschulsky)^65^. However, studies have concluded that *p*-coumaric acid and ferulic acid have negative effects on the performance of the grain aphid (*S*. *avenae*)^66^. The discrepancy among these studies might be due to the feeding guilds or food habits of herbivores, such as specialist and generalist responding differently to plant secondary chemistry^67–69^, reflecting different coevolution between insects and plant defence. In this study, genistin acted as an antifeedant of both the pea aphids and cowpea aphids, which had been demonstrated to negatively affect stinkbugs (*Nezara viridula* and *Piezodorus guildinii*) and whitefly (*Bemisia tabaci*)^70,71^. They all seem to imply that genistin may be important secondary compounds conferring resistance to many insects that belong to different feeding guilds. Our study also indicated that *p*-coumaric acid did not play a key role in responses of the cowpea aphids feeding to elevated O_3_, though *p*-coumaric acid acted as their feeding stimulant. This may be because many compounds coexist in plant leaves and interactions among them may alter the effects of a single chemical^62^.

In contrast to studies suggesting that elevated CO_2_ favours aphid phloem feeding, such as pea aphid fed on *Meidicago truncatula* and green peach aphid fed on *Nicotiana attenuata*^72–74^, our research demonstrated that elevated CO_2_ discouraged the pea aphids feeding at the reproductive stage, though favoured the cowpea aphids feeding at the vegetative stage. These results imply that the responses of aphids or even aphid-plant to elevated CO_2_ are species-specific. The heterogeneous responses have been widely reported^75^, however, the underlying mechanism is very complex. Our study suggests that the differential responses of two species of aphids to various compounds (genistin, ferulic acid, and *p*-coumaric acid) may be one reason. Indeed, studies have also shown that the aphid species or genotypes differentially respond to the same secondary compounds, such as thymol, hydroquinone, and alkaloid^38,76^, but more efforts are needed to explain the heterogeneous responses of aphids to atmospheric changes.

The performance of aphids can be decreased, increased, or unchanged under elevated O_3_, depending on the duration and concentration of O_3_ exposure and the age of the exposed plants^32,77^. However, our results showed that elevated O_3_ decreased the feeding efficiency of aphids at both plant developmental stages, except for no impact on phloem feeding of the pea aphids at the reproductive stage. Furthermore, the feeding efficiency of two species of aphids was inhibited under simultaneously elevated CO_2_ and O_3_, indicating that elevated CO_2_ did not completely offset the negative effects of elevated O_3_. This finding is in contrast to previous studies that elevated CO_2_ ameliorates the negative impact of elevated O_3_ on herbivores^19,78^. A possible explanation is that those studies use leaf-chewing herbivores and use trees as the host plants, while we use phloem-feeding insects and use herbage as the host plants. The SoyFACE experiments have demonstrated that elevated O_3_ has no impacts on soybean aphid (*Aphis glycine*) numbers, and that the effects of simultaneously elevated CO_2_ and O_3_ on aphid are similar to that of elevated CO_2_ alone^79^. Thus, the responses of plant growth forms and feeding guilds to atmospheric changes seem to be heterogeneous.

The decreased time the pea aphids and cowpea aphids spent on phloem feeding under simultaneously elevated CO_2_ and O_3_ may result in reduced direct damage to the plants. In addition, the aphid feeding activities can also be used to predict indirect damage caused by transmitting plant virus. The piercing-sucking mouthparts of aphids facilitate the delivery of virions into plant cells without causing irrevocable damage^80^. Among a series of aphid feeding activities, the potential drops (pd) and phloem feeding (E) are relevant to virus transmission^81^. Elevated CO_2_ and O_3_ significantly influenced the time aphids spent on potential drops (pd) and the total number of potential drops (pd), which in turn altered the transmission of stylet-borne viruses by aphids. However, more evidence is needed to demonstrate how the loss of plant productivity caused by virus transmission will be altered under future atmospheric changes.

In summary, elevated CO_2_ and O_3_ have the potential to affect aphid feeding behaviors via the alteration of plant secondary metabolites, and the responses of aphids to climate changes depend on aphid species and plant developmental stage. This study has generated several significant findings. First, it provides evidence that the heterogeneous responses of aphids to atmospheric changes may result from the differential responses of aphids to the chemicals. Second, direct damage and population outbreaks of aphids may be decreased under future atmospheric conditions due to the reduced efficiency of phloem ingestion under simultaneously elevated CO_2_ and O_3_. Finally, and perhaps most importantly, the responses of aphids to elevated CO_2_ and O_3_ alone or in combination are different and vary with plant developmental stage, suggesting that multi-factor and long-term research is needed. More research is needed to further elucidate the mechanisms underlying the effects of elevated CO_2_ and O_3_ on herbivores and the role of plant secondary metabolites in the adaption of aphids to future atmospheric environments.

## Methods

### Treatments under different CO_2_ and O_3_ concentrations

The study was performed from March to July 2015 in 12 octagonal open-top chambers (OTCs) at the Observation Station of the Global Change Biology Group, Institute of Zoology, Chinese Academy of Sciences in Xiaotangshan County, Beijing, China (40°11′N, 116°24′E). The atmospheric CO_2_ and O_3_ concentration treatments were as follows: current atmospheric CO_2_ and O_3_ concentrations (CK, 400 μL/L CO_2_ and 35 nL/L O_3_), elevated CO_2_ concentration (ECO_2_, 750 μL/L CO_2_), elevated O_3_ concentration (EO_3_, 70 nL/L O_3_), and simultaneously elevated CO_2_ and O_3_ concentrations (ECO_2_ + O_3_, 750 μL/L CO_2_ and 70 nL/L O_3_). Three blocks were used for CO_2_ and O_3_ treatments, and each block contained four OTCs, one OTC with ambient atmospheric CO_2_ and O_3_ concentrations, one OTC with an elevated CO_2_ concentration, one OTC with an elevated O_3_ concentration, and one OTC with elevated CO_2_ and O_3_ concentrations. CO_2_ and O_3_ concentrations in each OTC were monitored and adjusted with an infrared CO_2_ analyser (Ventostat 8102; Telaire Company, Goleta, CA, USA) and O_3_ analyser (Aeroqual, series 200, New Zealand), respectively, once every minute to maintain relatively stable CO_2_ and O_3_ concentrations. The OTCs were ventilated with air daily from 8:00 am to 5:30 pm.

### Aphids and host plants

The pink pea aphid (*A*. *pisum*) and the cowpea aphid (*A*. *craccivora*) were collected from alfalfa (*M*. *sativa*). The nymphal instars from the same parthenogenetic aphid female were reared on alfalfa with 14 h light (25 °C): 10 h dark (22 °C) in photoclimate chambers (Safe PRX-450C, Ningbo, China).

The alfalfa cultivar ‘Algonquin’ was purchased from the Chinese Academy of Agricultural Sciences. The ‘Algonquin’ seeds were sown in sterilized soil and watered every 4 days. After the seedlings had grown in sterilized soil for 2 weeks, they were transplanted into plastic pots (17 cm diameter and 24 cm height) and placed in the OTCs. Pot placement was re-randomized within each OTC once per week. No chemical fertilizers or insecticides were used. After the plants were fumigated for 3 weeks (vegetative stage) or 8 weeks (reproductive stage), they were used for the assays described in the following sections.

### Plant growth traits

Six plants per OTC (18 plants for each treatment and 72 plants in total) were randomly selected for measurement of growth traits. The leaf chlorophyll content was determined with a Minolta SPAD-502 plus (Konica Minolta Sensing Inc., Osaka, Japan). The leaf net photosynthetic rate was determined with a Li-Cor 6400 gas exchange system (Li-Cor Inc., Lincoln, NE, USA) between 9:00 hours and 12:00 hours. These plants were observed every day for flowering and podding after they had grown for 2 months. The remaining 12 plants per OTC (six for the vegetative stage, six for the reproductive stage, and 144 plants in total) were harvested for measurement of the biomass. The shoots and roots of each plant were collected, oven-dried (50 °C) for 48 h, and weighed.

### Aphid feeding behaviors

Seven plants per OTC (21 plants for each treatment and 84 plants for each aphid) were randomly selected as host plants at the vegetative stage to evaluate aphid feeding behaviors using the electrical penetration graph (EPG) technique. Another 84 plants were also randomly selected at the reproductive stage. The principle of EPG was introduced by Tjallingii and Hogen-Esch^82^. Eight plants were placed in a Faraday cage to avoid noise and interference. Each plant was infested with one apterous adult aphid, and its feeding behavior was recorded for 8 h. The aphids were starved for 10 h before the test. Two eight-channel amplifiers simultaneously recorded 16 individual aphids on separate plants (four plants per treatment). Twenty-one biological replicates were included for each treatment. The feeding waveforms in this study were scored according to Tjallingii and Hogen-Esch^82^: nonpenetration (np), stylets are outside the plants; pathway (C), mostly intramural probing activities between mesophyll or parenchyma cells; phloem feeding (E), aphids are injecting watery saliva into the sieve element and ingesting the phloem sap; potential drops (pd), aphids briefly puncture cells during plant penetration.

### Plant secondary metabolites

The chemical analysis was determined according to Oleszek and Stochmal^83^ and Nour *et al*.^84^ with some modification. Freeze-dried leaves were ground into a fine powder. For a typical extraction, approximately 50 mg samples were soaked with 1.5 mL of 70% aqueous MeOH for 1 h in a 60 °C water bath. The extract was centrifuged at 12,000 rpm for 15 min, and the supernatant was filtered with a 0.22 μm filter. The samples were stored in a −20 °C freezer until chemical analysis. Using high-performance liquid chromatography (HPLC), we analysed 12 phenolic compounds: phenolic acids, including chlorogenic acid, caffeic acid, cinnamic acid, *p*-coumaric acid, and ferulic acid; flavonoids, including rutin, luteolin, apigenin, kaempferol, and quercetin; and isoflavones, including genistein and genistin. Among these compounds, chlorogenic acid, caffeic acid, and cinnamic acid were not detected in the alfalfa leaves. Determination of compounds was performed on a Waters system with a diode array detector. Chromatograms were registered and integrated at 280, 350, and 254 nm for phenolic acid, flavonoids, and isoflavone, respectively. The mobile phase consisted of 1% H_3_PO_4_-AcN (a linear gradient of 15–100% AcN) with a flow rate of 1 mL/min for 60 min. Compounds were identified by comparing retention times to those of authentic standards.

### Bioassays with pure compounds

According to the chemical analysis, elevated CO_2_ and O_3_ had significant impacts on foliar rutin, genistin, ferulic acid, *p*-coumaric acid, genistein, and apigenin contents of alfalfa (Table 2). As aphid feeding behaviors were altered under elevated CO_2_ and O_3_, and as aphid feeding activities were significantly related to genistin, ferulic acid, *p*-coumaric acid, and apigenin (Table 4), we performed bioassays to test whether the four secondary compounds affected the aphid feeding. All test compounds were commercially available products.

For the bioassay, differing from the *in vitro* detached leaves and artificial diets^73,85^, we applied the pure compounds to the living plant leaves to provide aphids with the most realistic feeding conditions. All the bioassays were performed on alfalfa plants in a greenhouse. Twenty plants were randomly selected for the experiment. The leaves in the same location were separately treated with 50 μL genistin, ferulic acid, *p*-coumaric acid, and apigenin solution. Five biological replicates were included for each treatment. Another five plants were selected as controls. The treated and adjacent systematic control leaves were collected to measure the concentrations of the four compounds at 24 h, 48 h, and 72 h after treatment. Contents of genistin, ferulic acid, *p*-coumaric acid, and apigenin were much higher in the treated leaves compared to the control leaves. The variation tendency remained similar at 24 h, 48 h, and 72 h (see Supplementary Table S1 online). Thus, we selected 24 h to assess the effects of compounds on aphid feeding behaviors using EPG as above.

### Statistical analysis

We analysed the univariate responses of the growth traits, secondary metabolite contents and aphid feeding activities with a split-plot design using the following model *Y*_*ijkl*_ = *b*_*i*_ + *C*_*j*_ + *O*_*k*_ + *CO*_*jk*_ + *e*_*ijk*_ + *D*_*l*_ + *CD*_*jl*_ + *OD*_*kl*_ + *COD*_*jkl*_ + *ε*_*ijkl*_. In this model, *b* represents block *i* = 3, *C* represents CO_2_ level *j* = 2, *O* represents O_3_ level *k* = 2, *e*_*ijk*_ represents the whole-plot error, *D* represents developmental stage *l* = 2, and *ε*_*ijkl*_ represents the sub-plot error. *Y*_*ijkl*_ represents the average response of block *i*, CO_2_ level *j*, O_3_ level *k* and developmental stage *l* (SAS 9.2, USA). Effects were considered significant when *P* < 0.05. LSD multiple range tests were used to separate means when ANOVAs were significant.
